# A Pilot Study of an Innovative Model of Care Delivery: Service Robot for Older People with Dementia in a Rural Community

**DOI:** 10.1093/geroni/igaa057.3402

**Published:** 2020-12-16

**Authors:** Kuan-Yuan Wang, Cheng-Sheng Chen, Hui-Mei Chen, I-Te Chen

**Affiliations:** Kaohsiung Medical University Hospital, Kaohsiung Medical University, Kaohsiung, Taiwan (Republic of China)

## Abstract

Background: The telehealth approach offers enhanced service delivery for older patients living in rural areas. Purpose: We conducted a pilot study to evaluate the feasibility of using the Zenbo robot to improve the quality of care of elderly individuals with dementia. Methodology: In this study, we developed a digital solution on service robots and smart devices, which can leverage the capacity of the user-friendly interactive interface and digital dialog system. A group of eleven volunteered older adults was selected for this study. To assess the likability and acceptance of the Zenbo, we conducted a one-on-one (robot vs human) pilot study in our long-term care stations. Each participant engaged in conversational interactions for five consecutive days and completed a survey of 12 questions about the experiences they had with the Zenbo, at the beginning and the end of the study respectively. Results: Subjects with lower GDS-15 scores have more positive attitudes toward the robot before the intervention. 27% of older adults had an increase in positive attitudes toward the interaction with the Zenbo robot. With the aid of teleoperated mobile robotic systems at home, the innovative service model can be achieved through telecommunication between primary health professionals or caregivers at remote locations and psychiatrists at the medical center to make the seamless care environment real. Conclusion: The IoT technologies can be used to assist physicians in switching from a hospital-centered model of care to a home-based service for older people with dementia. It merits more future clinical trials and usability tests.

